# Spermatic cord torsion: a retrospective analysis

**DOI:** 10.31744/einstein_journal/2023AO0238

**Published:** 2023-06-06

**Authors:** Renan Murata Hayashi, Alexandre Kyoshi Hidaka, Felipe Placco Araújo Glina, Khalil Smaidi, Cristiano Linck Pazeto, Fabio José Nascimento, Willy Baccaglini, Pedro Henrique Borba Leite, Antonio Corrêa Lopes, Sidney Glina

**Affiliations:** 1 Centro Universitário FMABC Santo André SP Brazil Centro Universitário FMABC, Santo André, SP, Brazil.

**Keywords:** Spermatic cord torsion, Emergencies, Testis, Orchiectomy

## Abstract

**Objective:**

To evaluate the time interval and possible delay in transportation to referral units for the treatment of testicular torsion.

**Methods:**

We retrospectively analyzed all cases of spermatic cord torsion surgically treated at a university hospital between January 2018 to December 2021. We evaluated the time intervals, including pain onset until the first presentation (D1), interhospital transference time (D2), pain onset until urological evaluation in a tertiary service (D3), urological evaluation until surgery (D4), and time from pain onset to surgical treatment (D5). We analyzed demographic and surgical data, orchiectomy rates, and time intervals (D1–D5). Torsions presented to the first medical presentation within 6h were considered early for testicular preservation.

**Results:**

Of the 116 medical records evaluated, 87 had complete data for the time interval analysis (D1 to D5) and were considered the total sample. Thirty-three had D1 ≤6h, 53 had D1 ≤24h (includes patients in the D1 ≤6h subgroup), and 34 had D1 >24h. The median time intervals of the total samples and subgroups D1 ≤6h, D1 ≤24h, and D1 >24h were D1 = 16h 42min, 2h 43min, 4h 14min and 72h, D2 = 4h 41min, 3h 39min, 3h 44min and 9h 59min; D3 = 24h, 6h 40min, 7h and 96h; D4 = 2h 20min, 1h 43min, 1h 52min and 3h 44min; D5 = 24h 42min, 8h 03min, 9h 26min and 99h 10min, respectively. Orchiectomy rates of the total sample, subgroups D1 ≤6h, D1 ≤24h, and D1 >24h were 56.32%, 24.24% (p<0.01), 32.08% (p<0.01), and 91.18% (p<0.01), respectively.

**Conclusion:**

Late arrival at the emergency department or a long interhospital transference time determined a large number of patients who underwent orchiectomy. Thus, public health measures and preventive strategies can be developed based on the data from this study aiming to reduce this avoidable outcome.

## Highlights

■ Less than half of the patients (37.93%) reached first medical unit within 6h of pain onset.

■ Patients with delayed first medical attendance showed higher orchiectomy rates.

■ A high delay in seeking assistance and considerable time spent on interhospital transport were observed.

■ Late presentation (D1) and interhospital transport (D2) had a negative impact on testis preservation.

**Figure:**
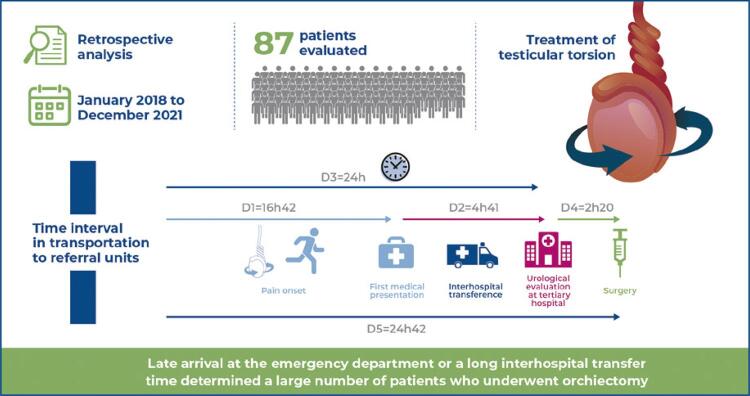

## INTRODUCTION

Acute scrotum is a urological urgency characterized by acute testicular pain. Spermatic cord torsion is one of the three most frequent forms of this condition, affecting 3.8 per 100,000 men up to 18 years of age annually, with a bimodal peak in the perinatal period (up to 1 year) and the pubertal period (12-16 years).^(1)^ It is clinically characterized by sudden and intense unilateral testicular pain in which the testes may be in an abnormal position, horizontally and elevated, with no cremasteric reflex, scrotal edema, or a negative Prehn reflex (no improvement in pain on elevation of the testicles). In some cases, the pain is accompanied by nausea and vomiting.

Clinical diagnosis can be complemented by imaging tests such as color Doppler ultrasound (US), high-resolution US, and scrotum scintigraphy.^(2-4)^ Although they provide information on testicular blood flow and have high sensitivity and specificity, they are examiner-dependent and are not always available to physicians in the emergency department. Therefore, its diagnosis must be clinical in the absence or delay in performing these tests.^(5)^

Currently, the resolution approach involves surgical exploration with spermatic cord distortion and bilateral orchidopexy. Orchiectomy is reserved when ischemia leads to irreversible damage or necrosis of the testicular parenchyma.^(6)^

Early surgical intervention, the time between the onset of symptoms and surgery, and the degree of torsion are determining factors for the rescue of the affected gonad. Currently, the testicular salvage rate is approximately 95% 6h after the onset of symptoms; however, in patients with a torsion time >24h, the probability of testicular death is >80%. In addition, twists greater than 360° are associated with a higher rate of testicular loss.^(7,8)^

Knowledge about this urological emergency in the general population is important for the search of early care at the onset of symptoms to avoid orchiectomy. For physicians, mastery of the management of this condition is essential for quick and accurate diagnosis, consequently favoring an increase in the testicular salvage rate.^(8)^

## OBJECTIVE

This study aimed to perform a retrospective epidemiological analysis of patients diagnosed with torsion of the spermatic cord between 2018 and 2021, analyzing the duration and possible delays regarding transportation to referral units in the treatment of testicular torsion.

## METHODS

We included cases of spermatic cord surgically treated at the tertiary referral service from January 2018 to December 2021.

**Figure 1:**
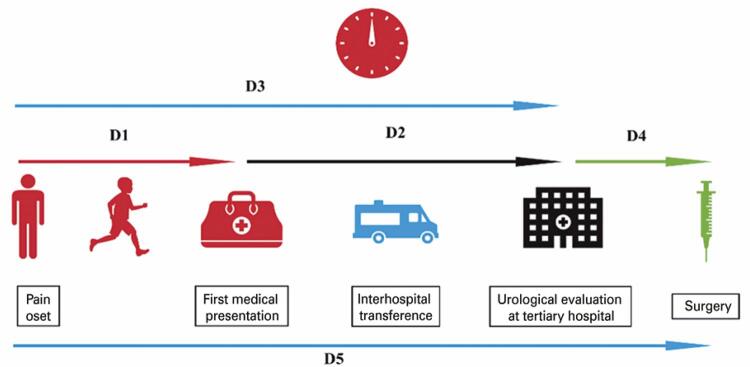
Patient´s timepoints to treatment

In the city, all patients with suspected testicular torsion are referred to a tertiary hospital. Generally, they come from other non-tertiary hospitals, where the first care is provided by a pediatrician or an emergency physician. When testicular torsion is suspected based on physical examination, these services send an application form to the city regulatory agency, which intend sends them to the only urology service in town. Upon arrival at the tertiary hospital, the urologist re-evaluates the patient, and if necessary, the surgical procedure is immediately indicated (Figure 1). When there is doubt regarding the diagnosis and viable time, Doppler Ultrasonography of the Scrotum and evaluation by a radiologist is performed. The surgical procedure is immediately indicated when there is a doubt or absence of radiological evaluation.

Owing to the need for interhospital transport, all time intervals were measured to assess their impact on treatment. The time interval between the onset of pain and first medical presentation was defined as D1. Interhospital transference time was D2, the time interval between the onset of pain and urological evaluation at the local tertiary hospital was D3, the interval between the urological evaluation and the beginning of the surgery was D4, and the interval between the onset of pain and the beginning of surgery (total time to treatment) was D5.

The data collected included the type of surgical procedure (orchiectomy or orchidopexy), testis positioning (horizontal or elevated), intraoperative testicular appearance (ischemic or necrotic), and degree of twist.

We calculated the orchiectomy rates for the total sample and subgroups according to the following intervals: D1 ≤6h, D1 ≤24h and D1 >24h. The D1 ≤24h subgroup also included patients with D1 ≤6h. It was considered as an early time for testicular preservation (TP) in cases that presented up to 6 h of the beginning of the symptoms.

The primary outcome was the time interval (D1–D5).The secondary outcomes were the orchiectomy rates of the total sample and subgroups according to the intervals: D1 ≤6h, D1 ≤24h and D1 >24h.

We used the χ^2^ test to compare categorical variables. The data were then examined for autocorrelation.

This study was approved by the local ethics committee of *Centro Universitário FMABC* (CAAE: 42394821.7.0000.0082; # 4.638.750).

## RESULTS

During the study period, 116 patients with a confirmed spermatic cord torsion were treated; however, only 87 of these had time stamps in their medical records to access the time intervals D1–D5 and were retained for the final analysis.

Thirty-three (37.93%) patients had time between the pain onset and the first medical presentation D1 ≤6h, 53 (60.92%) had D1 ≤24h, and 34 (39.08%) had D1 >24h, respectively. When we evaluated all the samples by their D3, only 22.22% arrived within 6 h of pain onset and 56.32% arrived within 24h of pain onset to the urologist.

Demographic data showed a median age of 14 years (range: 11–46 years), with a higher incidence between 13 and 14 years of age. The right testicle was more frequently affected (60.92%) (Table 1).

**Table 1:** Demographic data

Variables	
Total patients, n (%)	87 (100)
Orchiectomy	49 (56.32)
Median age (y) range	14 (11-46)
Torsion laterality, n (%)	
Right testis	53 (62.93)
Left testis	32 (36.78)
Bilateral	2 (2.30)
Race, n (%)	
Brown	42 (48.28)
White	35 (40.23)
Black	3 (3.45)
No information	7 (8.05)
Mean BMI (kg/m^2^)	21.30 (n=19)
Symptom profile, n (%)	
Testicular pain	87 (100.00)
Nausea and/or vomiting	15 (17.24)
Scrotal edema	53 (60.92)
Absence of cremasteric reflex	7 (8.05)
Testicular hardening	30 (34.48)
Testis positioning, n (%)	
Horizontalized	34 (39.08)
Elevated	22 (25.29)
Testicular surgical aspect, n (%)	
Ischemia	39 (44.83)
Necrosis	27 (31.03)
Torsion degrees, n (%)	63 (72.41)
>360º	40 (57.47)
<360º	23 (26.44)

BMI: body mass index.

The degree of torsion was reported in 63 of the 87 charts. Considering only these 63 charts and the variable surgical procedures and degree of torsion, the orchiectomy rate with degree of torsion ≥360˚ was 72.00% [p=0.0036], while the orchiectomy rate with degree of torsion <360˚ was 23.08% [p=0.0036].

The medians time intervals D1, D2, D3, D4, and D5 and their subgroups are shown in table 2.

**Table 2:** Subgroups *versus* time intervals comparison

Subgroups *versus* time intervals	D1	D2	D3	D4	D5
Total sample (n=87)	16h 42min	4h 41min	24h	2h 20min	24h 42min
D1 <6h (n=33)	2h 43min	3h 39min	6h 40min	1h 43min	8h 03min
D1 <24h (n=53)	4h 14min	3h 44min	7h	1h 52min	9h 26min
D1 >24h (n=34)	72h	9h 59min	96h	3h 44min	99h 10min

D1: Time interval between pain onset until first medical presentation; D2: Time interval of interhospital transference; D3: Time interval between pain onset and urological evaluation at tertiary hospital; D4: Time interval between urological evaluation at our service and surgery beginning; D5: Time interval between pain onset and surgery beginning (overall time to treat).

**Figure 2:**
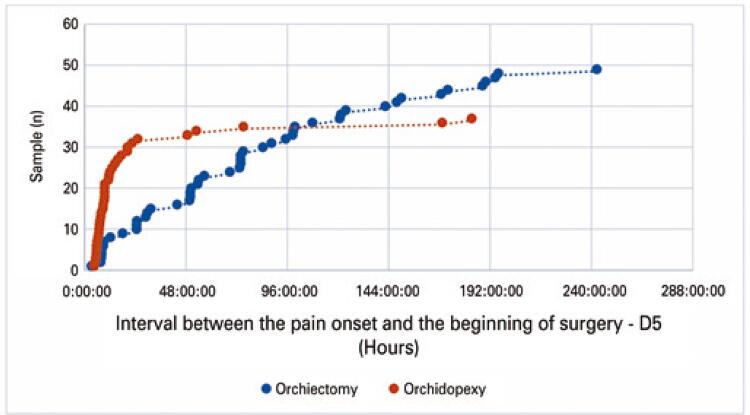
Surgical procedure *versus* total treatment time (D5)

The orchiectomy rate in the total sample was 56.32%. In the subgroups of patients with D1 ≤6h, D1 ≤24h and D1 >24h, the orchiectomy rates found were 24.24% (p<0.0001), 32.08% (p<0.0001), and 91.18% (p<0.0001), respectively. It should be noted that the 8.82% of orchidopexies in the D1 >24h subgroup were performed in patients who, because they had an atrophic contralateral testicle or only one testicle, chose to keep the organ in order to maintain the hormonal status. Regarding total time to treat (D5) and the surgical procedure adopted, 81,63% of the orchiectomies had D5 >24h. Figure 2 compares the surgical procedure (orchiectomy or orchidopexy) with the total treatment time (D5).

## DISCUSSION

Surgical resolution of testicular torsion is a race against time. It is estimated that spermatic cord torsion treated within 6h has a testicular salvage rate of approximately 95%. For patients with torsion time >24h, the probability of testicular death is greater than 80%.^(8)^

Although most patients (60.92%) arrived at the first medical presentation within 24h of the pain onset, the subgroup with D1 ≤6h represented only 37.93% of the total sample. Therefore, less than half of the patients presented with testicular preservation in the first consultation, indicating that the majority of the population was unaware of the severity of the clinical signs and consequences of delay in seeking medical care.

Despite the urgency of this condition, cases with more than 24h may have a delayed time to treat, associated with a salvage rate inferior to 10–20%.^(7)^Therefore, a separate analysis of the cases within 24h of pain onset (D1 <24h subgroup) was conducted. A median D2 value close to 4h was observed. This elevated time did not favor testicular preservation.

Preece et al. conducted a single-center retrospective study in 2017 and reported that 85% of patients reached their first medical presentation within 24h. They compared individuals who were directly admitted to the service with those who required interhospital transport. It was observed that those who required transference had almost twice the rate of orchiectomy than those who arrived directly at the study hospital (30.4% *versus* 15.2%, p=0.129).^(9)^ In 2010, Bayne et al. advocated that the distance between the hospital and delay time related to hospital transference may postpone the treatment time.^(10)^ In a recent systematic review and meta-analysis, Kwenda et al. found that the time between the onset of pain and the first evaluation was more important than the transport time and processing time in emergency rooms.^(11)^ Thus, the data obtained in the present study corroborate the findings mentioned above.

The time interval between the onset of pain and the urology evaluation at our local hospital (D3) showed that only 22.22% of patients arrived at the urology unit in <6h. This finding is not favorable for testicular preservation in our sample. The late presentation time for the first evaluation associated with a prolonged interhospital transport time was the two main factors for the delay in the observed treatment in this study.

The median time intervals between the urological evaluation until the beginning of the surgery (D4) also affected the total time for surgical treatment; however, this time was due to internal processing from admission to transport to the operating room and anesthesia. We believe that this process can be improved by analyzing the internal processing steps.

When the individual interval between the beginning of pain and the beginning of the surgery (D5) was compared to the surgical procedure adopted for each case (Figure 2), it showed an orchiectomy rate after 24h of torsion similar to that demonstrated by Visser et al.^(8)^

For patients with D1 >24h, due to the high rate of testicular loss, one should focus on population awareness campaigns to avoid this outcome, as proposed by Visser et al. in 2003.^(8)^

In the surgical site, the surgeon performed orchiectomy based on the ischemic or necrotic aspect of the testis. The 24.24% orchiectomy rate in the D1 ≤6h group reflects a high rate in this part of the sample, leading us to question the over-indication of orchiectomy in our service. To solve this bias, we started a prospective evaluation of orchidopexy and tunica vaginalis retail in all blue or purple testes within 24h of pain onset in October 2020. Future studies will be conducted to resolve this problem.

Doppler ultrasound was performed for <30 minutes in both locations and did not affect the overall result. The second US was performed immediately upon admission to our hospital with no delay in treatment. The surgical procedure was performed immediately in the US room. We could not conclude if this was a critical factor of probable delay to treatment, as some cases within 6h immediately underwent orchiectomy and others within 24h. Therefore, this analysis was excluded from the final study.

It is important that emergency services be instructed so that the arrival of patients with testicular pain can be seen as an emergency and care should be brief.

This retrospective study had several limitations. In D2, the internal processing of the other units, requests for evaluation and response from the urologist on call, waiting time until transport availability, transport time, and time until opening the care form at the tertiary hospital were included. We measured the D2 interval by measuring the time between the file opening in the primary emergency room and the file opening in our tertiary hospital. There may be some collection bias at this point, which should be better elucidated through internal assessment of the primary care unit, for which data were not available for this analysis. Prospective observational and individualized studies can provide more data on the processes of care and interhospital transport. The analysis of our sample was limited by the small number of eligible cases.

## CONCLUSION

The time of the first medical presentation and interhospital transport time directly affected the time of surgical treatment for spermatic cord torsion. Public health measures must be designed to reduce these avoidable outcomes. Future preventive strategies can be developed based on the data from this study.
